# Effect of radiation therapy on cerebral cortical thickness in glioma patients: Treatment-induced thinning of the healthy cortex

**DOI:** 10.1093/noajnl/vdaa060

**Published:** 2020-05-21

**Authors:** Steven H J Nagtegaal, Szabolcs David, Tom J Snijders, Marielle E P Philippens, Alexander Leemans, Joost J C Verhoeff

**Affiliations:** 1 Department of Radiation Oncology, University Medical Center, Utrecht, The Netherlands; 2 Image Sciences Institute, University Medical Center, Utrecht, The Netherlands; 3 Department of Neurology, University Medical Center, Utrecht, The Netherlands

**Keywords:** cerebral cortex, cortical thickness, glioma, radiation therapy

## Abstract

**Background:**

With overall survival of brain tumors improving, radiation induced brain injury is becoming an increasing issue. One of the effects of radiation therapy (RT) is thinning of the cerebral cortex, which could be one of the factors contributing to cognitive impairments after treatment. In healthy brain, cortex thickness varies between 1 and 4.5 mm. In this study, we assess the effect of RT on the thickness of the cerebral cortex and relate the changes to the local dose.

**Methods:**

We identified 28 glioma patients with optimal scan quality. Clinical CTs and MRIs at baseline and 1 year post-RT were collected and coregistered. The scans were processed via an automated image processing pipeline, which enabled measuring changes of the cortical thickness, which were related to local dose.

**Results:**

Three areas were identified where significant dose-dependent thinning occurred, with thinning rates of 5, 6, and 26 μm/Gy after 1 year, which corresponds to losses of 5.4%, 7.2%, and 21.6% per 30 Gy per year. The first area was largely located in the right inferior parietal, supramarginal, and superior parietal regions, the second in the right posterior cingulate and paracentral regions, and the third almost completely in the right lateral orbital frontal region.

**Conclusions:**

We have identified three areas susceptible to dose-dependent cortical thinning after radiation therapy. Should future prospective studies conclude that irradiation of these areas lead to cognitive decline, they need to be spared in order to prevent this debilitating consequence of treatment.

**Key Points**

- The cerebral cortex is susceptible to dose-dependent cortical thinning.
- Three distinct areas of cortical thinning after radiation therapy were observed.

**Importance of the Study**

Cognitive decline after cranial radiotherapy is a debilitating consequence of the current treatment for brain tumors. Next to damage to the white matter and hippocampus, thinning of the cerebral cortex is one of the hypotheses of its origin. Several papers have already found that this phenomenon does indeed occur after radiotherapy, but high-quality evidence remains lacking. In this work, we analyzed high-resolution MRI images from glioma patients before and after radiotherapy. We observed dose-dependent cortical thinning after treatment in three separate areas of the cortex, with thinning rates corresponding to aging by several years. We advise optimization of current brain sparing radiation techniques in order to reduce the cognitive sequalae of treatment for brain tumors. Future directions for research include linking these changes to cognitive outcome in order to determine the clinical implications.

Radiation therapy (RT) plays an important part in the treatment of brain malignancies, both for primary and metastatic disease. It comes, however, with the unfortunate consequence of radiation-induced brain injury. One of the major and most debilitating symptoms of this phenomenon is progressive cognitive decline, which occurs in 50%–90% of patients undergoing RT to the brain.^1,2^ It most likely has a multifactorial origin, with roles for vascular damage, demyelination, and neuronal dysfunction.^1–3^ Morphological changes in the hippocampus, white matter, and cerebral cortex related to radiation-induced brain injury can be seen on routine imaging^4–8^ (see Figure 1), and specialized software allows for accurate measurement of these changes.^9–12^ Interest in the effect of RT on cortical thickness and volume has increased in the recent years, but high-quality evidence remains sparse. In a recent review,^13^ the current knowledge was assessed, and two papers showing cortical thinning after RT were found. Karunamuni et al. were the first to show dose-dependent cortical thinning after RT in 15 glioma patients.^14^ Seibert et al. went on to examine brain regions associated with cognitive function and found these to be susceptible to radiation-induced damage.^15^

**Figure 1.**
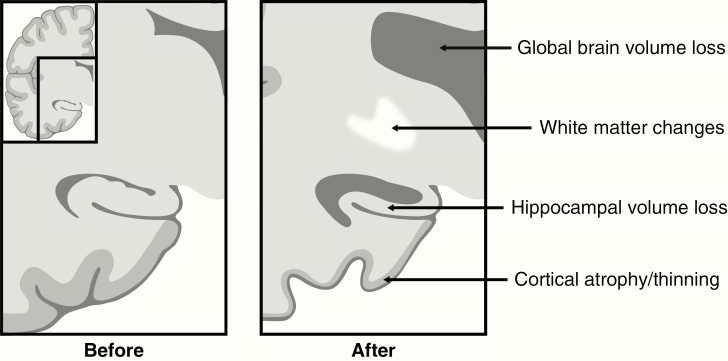
Previously reported radiological changes in the postradiation therapy brain related to cognitive changes.

Changes in cortical thickness have been linked to cognitive decline, suggesting that thinning of the cortex has a role in the etiology of radiation-induced cognitive impairments.^16,17^ Therefore, it would be of interest to assess whether RT does indeed cause thinning of the cortex and if this is related to the administered dose. Identification of areas susceptible to radiation-induced damage may encourage sparing of these areas. In this study, we wish to add to the existing evidence by assessing the effect of radiation therapy on the thickness of the cerebral cortex and relating the changes to the local dose.

## Methods

### Patient Selection and Data Collection

We retrospectively identified patients who were treated with RT for newly discovered grade II–IV glioma at the Department of Radiation Oncology of our institution in 2016 and 2017. Patients were eligible for inclusion when the following criteria were met: treatment planning CT and MRI present and of sufficient resolution; survival of at least 270 days after RT; and at least 1 follow-up MRI between 270 and 360 days after RT present and of sufficient resolution. Clinical MRI and CT scans made for RT treatment planning were extracted from patient records, along with all follow-up MRIs and clinical and demographic characteristics. Informed consent for this retrospective study was waived by our institutional review board (#18/274).

### Image Acquisition

For every patient, the pre-RT CT and MRI were collected, as well as all available follow-up MRIs. MR images were acquired on a 3T Philips Ingenia scanner (Philips Medical Systems) as part of routine clinical care. T1-weighted MR images were acquired with a 3D turbo-spin echo (TSE) sequence without gadolinium enhancement with the following parameters: TR = 8.1 ms, TE = 3.7 ms, flip angle = 8°, 213 continuous axial slices without gap, matrix: 207 × 289, voxel resolution 1 × 0.96 × 0.96 mm. The planning CT scans were acquired on a Brilliance Big bore scanner (Philips Medical Systems), with a tube potential of 120 kVp, with the use of a matrix size of 512 × 512 and 0.65 × 0.65 × 3.0 mm voxel size.

### Image Processing

**Figure 2.**
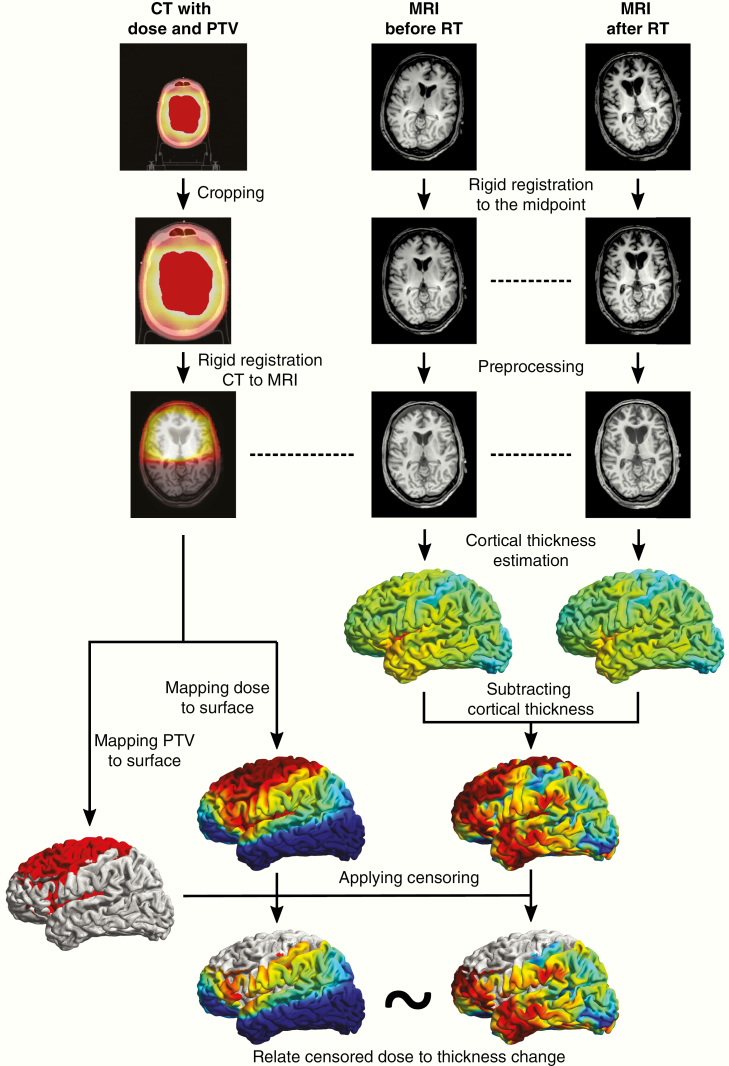
Image processing pipeline. PTV = planned target volume, RT = radiation therapy.

All imaging data was processed with Statistical Parametric Mapping,^18^ Computational Anatomy Toolbox (CAT12),^19^ and in-house algorithms developed in MATLAB (Mathworks). Image processing was done according to our own previously published criteria.^13^ A graphical overview of the image processing pipeline is shown in Figure 2. More detailed methods can be found in Supplementary Appendix 1.

In brief, the cropped CT image with the associated dose and planning target volume (PTV) maps was registered to the T1 MR images. This step resulted in the CT image and the MRIs being in the same space. Next, the rigidly coregistered T1s were processed with CAT12’s segmentation pipeline. If the residual damage to the tumor area (e.g., edema, surgical scarring, and tumor bed) extended beyond the PTV in either the baseline or the follow-up images, then the affected subject was removed from the analysis to prevent tissue misclassification.

Cortical thickness and central surfaces were estimated with CAT12 by means of the fully automated projection-based thickness estimation method. Dose maps and PTV maps (dilated by 3 mm and smoothed) were mapped to the template surface, meaning that all individual features (cortical thickness, dose, and PTV mask) were mapped to the same template surface. The within-subject difference in cortical thickness was calculated by subtracting the baseline and the follow-up cortical thickness surfaces in every vertex (a vertex being analogous to a voxel but on a surface). In every subject, the cortical thickness difference and the dose maps were censored with the surface-resampled PTV maps to avoid spurious thickness–dose relations, which may originate from false thickness estimation around the tumor. This resulted in dose and cortical thickness change maps, in which the tumor bed and tumor scar was censored and which could be used for further analysis.

### Statistical Analysis

Vertexwise statistical comparison of cortical thickness change and dose correlation was carried out with a permutation test with 10 000 iterations performed with the permutation analysis of linear models (PALM) toolbox in Matlab.^20–22^ Significance of a correlation was set at *P*_corr_ < 0.05, using family-wise error rate (FWER) adjustment to correct for multiple comparisons. Age at the time of the diagnosis and sex of the patients were included as nuisance regressors. Multiple effect size measures and the yearly rate of change per dose (in %/Gy/year) are reported to grade the practical significance of the results.^23–25^

To test whether chemotherapy has an effect on the dose–thickness relation, a sensitivity analysis was performed, in which we corrected for chemotherapy type (chemotherapy vs no chemotherapy). By adding this variable to the nuisance regressions of the permutation test performed in the primary analysis, we assessed whether this would lead to different results. Similar to the primary analysis, 10 000 iterations were performed with the PALM toolbox using FWER adjustment to correct for multiple comparisons.

## Results

### Participants

Of all patients who underwent RT for glioma between 2016 and 2017, 28 fulfilled our inclusion criteria and were selected for further analysis. A flow chart of study inclusion is shown in Supplementary Figure S1. Extensive cerebral edema after RT outside the PTV on the baseline imaging meant exclusion from further analysis in three patients. Baseline characteristics are shown in Table 1.

**Table 1.** Baseline characteristics of included patients

	*N* (total *n* = 28)
Age (mean; SD)	49 (±15)
Sex	
Male	17 (60.7%)
Female	11 (39.3%)
WHO grade	
II	11 (39.3%)
III	7 (25%)
IV	10 (35.7%)
Tumor type	
Astrocytoma	13 (46.4%)
Oligodendroglioma	3 (10.7%)
Mixed	1 (3.6%)
Ganglioglioma	1 (3.6%)
Glioblastoma	10 (35.7%)
Prescribed dose	
28 × 1.8 = 50.4 Gy	11 (39.3%)
30 × 1.8 = 54 Gy	2 (7.1%)
30 × 20 = 60 Gy	15 (53.6%)
Chemotherapy	
None	4 (14.3%)
Temozolomide	20 (71.4%)
PCV	4 (14.3%)

PCV, procarbazine, lomustine, and vincristine.

### Cortical Thickness

Mean cortical thickness at baseline and follow-up, as well as relative and absolute differences between the two are shown in Supplementary Figure S2. Maps of maximum, mean, and minimum dose to the surface are shown in Supplementary Figure S3.

**Figure 3.**
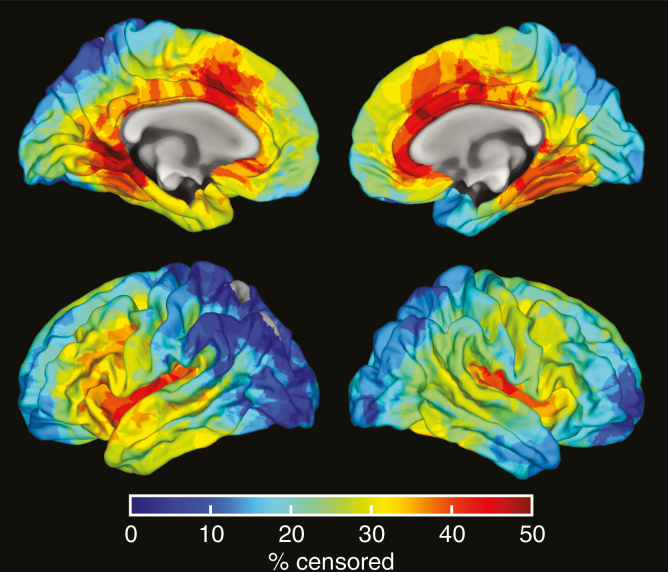
Heatmap showing the number of censored areas in the cortex.

**Figure 4.**
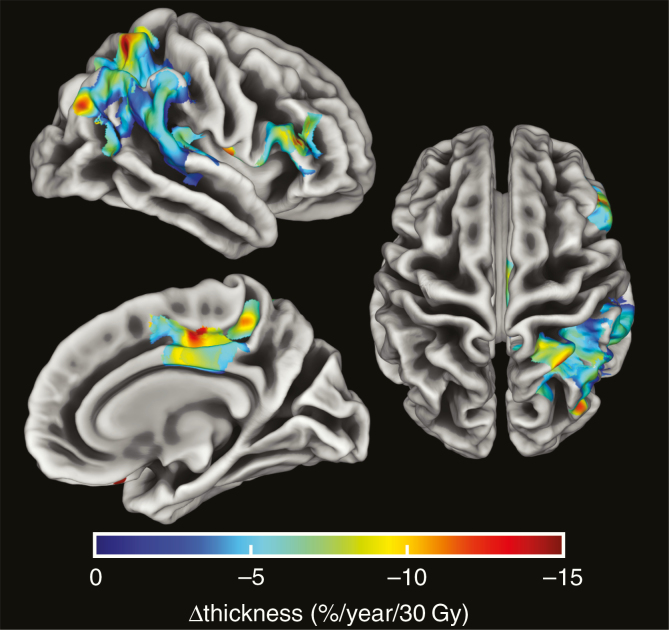
Areas of significant dose-dependent cortical thinning in percentage of change/year/30 Gy.

Three areas were identified where significant dose-dependent thinning occurred, with thinning rates of 5, 6, and 26 μm/Gy. The first area was largely located in the right inferior parietal, supramarginal, and superior parietal regions, the second in the right posterior cingulate and paracentral regions, and the third almost completely in the right lateral orbital frontal region. The precise location of the areas can be seen in Figure 3, and the details per region are shown in Table 2. A map showing the spatial distribution of censorings in the cortex is shown in Figure 4. A map of corrected *P*-values of the relationship between dose and cortical thinning is shown in Supplementary Figure S4.

**Table 2.** Areas of significant dose-dependent cortical thinning in the right hemisphere

Cluster	Cluster size (no. of vertices)	Mean cortical thickness change (μm/Gy/year)	Mean cortical thickness change (%/Gy/year)	*P*-value*	Corresponding atlas regions**
1	23 424	−5	-0.18	0.009	Inferior parietal (24.3%)
					Supramarginal (20.2%)
					Superior parietal (19.8%)
					Superior temporal (9.5%)
					Pars triangularis (5.4%)
					Pars opercularis (5.4%)
					Postcentral (3.4%)
					Transverse temporal (3.4%)
					Insula (2.1%)
					Precentral (2.31%)
					Rostral middle frontal (1.8%)
					Bank of STS (2.4%)
2	4876	−6	−0.24	0.023	Posterior cingulate (43.4%)
					Paracentral (31.8%)
					Precuneus (12.5%)
					Superior frontal (12.3%)
3	209	−26	−0.72	0.046	Lateral orbital frontal (98.0%)
					Medial orbital frontal (2.0%)

*Corrected for multiple testing with family-wise error rate adjustment.

**According to Desikan–Killiany brain atlas.^28^

A sensitivity analysis was done to test whether chemotherapy has an additional effect on the dose–thickness relation. The results are shown in Supplementary Table 1. The same areas with a significant result were found as in the primary analysis, with similar thinning rates and *P*-values. Only small variations in cluster size and corresponding atlas regions were present, resulting in region 2, as found in the primary analysis, now being identified as two separate regions with the same cortical thinning rate of −6 μm/Gy.

## Discussion

In this study, we examined the change in cortical thickness after RT of the brain. We found that there are three areas in the right hemisphere that show significant dose-dependent cortical thinning. The effect of RT on these cortical areas is in the same order of magnitude as aging by several years, in some regions even up to a decade.^26,27^

Cortical thickness and volume change have been examined before in several other studies with varying levels of evidence,^13^ with the most complete and robust analysis presented in the papers by Karunamuni et al. and Seibert et al.^14,15^ Karunamuni et al. analyzed 15 glioma patients before and after RT and found dose-dependent cortical thinning with rates of 1.3 μm (up to 34.6 Gy) and 7.2 μm/Gy (above 34.6 Gy). Seibert et al found several brain regions (prespecified according to the Desikan–Killiany brain atlas^28^), which showed a decrease in cortical thickness with increased dose. Two of these regions, the inferior parietal and superior temporal, overlap with the areas found in our analysis and showed thinning rates from 2.3 and 4.4 μm/Gy, respectively

The areas showing dose-dependent identified in the current work are comprised of several functional cortical areas involved in cognitive processes.^29^ The supramarginal, inferior parietal, and superior temporal gyri, the latter two also identified by Seibert et al.,^15^ are part of Wernicke’s area. This area is involved in both language comprehension and speech production, and damage to it can lead to aphasia.^30^ The first cluster also contained the superior parietal lobule, which is a somatosensory association area, involved in visuospatial coordination and working memory.^29,31^ The posterior cingulate cortex is well connected, being part of both the hippocampal and limbic systems, and is associated with memory and emotion.^29^ Finally, the lateral orbital frontal gyrus is located in the ventromedial prefrontal cortex. Impulse control is in part mediated by this region as a result of its connection to the amygdala.^29^

Loss of cortical thickness is part of the normal aging process.^32^ However, accelerated cortical thinning (with rates comparable to our findings) has also been observed in neurodegenerative diseases, such as Alzheimer’s disease^33,34^ and Parkinson’s disease,^35^ as well as other disorders, such as depression.^36,37^ An association between cortical thinning and cognitive impairments has also been found, which suggests that cortical thinning is one of the mechanisms underlying the impairments observed after radiation therapy.^16,17^

The pathophysiological process that leads to a diminished cortex remains unknown. Observed differences in the post-RT brain include phagocytosis of healthy and damaged neurons though activated microglia,^38^ loss of glial cells,^39^ and demyelination through vascular damage.^1,2,40^ The exact mechanism behind cortical thinning is most likely a combination of some or all of these radiation effects.

As mentioned before, the changes in the cerebral cortex are not the only effects that are seen after RT (see Figure 1). Deep gray matter structures, including the hippocampus^41^ and the amygdala,^42^ were already shown to have a susceptibility to dose-dependent volume loss after RT. These structures, as well as the cerebral cortex, may in the future be considered as organs at risk to be avoided by the radiotherapy planning system.

Due to advances in therapy, mortality of cancer has decreased over the last decades, which means that the number of cancer survivors has increased; ^43^ although gliomas can rarely be treated curatively, the therapeutic advances have led to improved overall survival for patients. Naturally, this is a desirable development, but it also means that long-term consequences of anticancer treatment have to be examined. If a treatment leads to excellent survival but also comes with serious chronic adverse effects, the consideration has to be made whether this is the optimal treatment. Patient-tailored decisions need to be made and patients need to be informed not only of the expected benefits of survival but also of the long-term consequences. As the prominent long-term effect of brain tumor treatment is cognitive decline, we need to be able to predict what certain therapies do to patients’ cognitive abilities.

In this work, we established an association between cortical atrophy and dose, while the clinically significant relation between changes in cortical thickness and the changes in the patients’ cognition remains unknown. Important to note is that neurophysiological investigations already revealed the connection between RT and cognition.^1,2^ The hypothesis that cortical thickness decrease relates to cognitive symptoms, just like in other brain diseases, such as Alzheimer’s disease, fits in this line of observations. As far as we know, no study has examined the relation between these three factors in a single prospective cohort, which seems to be the reasonable follow-up to understanding this debilitating phenomenon.

Additionally, the feasibility of cortical sparing should be examined, the possibility of which has already been shown.^44,45^ If areas of dose-dependent cortical thinning are found that affect the cognitive outcomes after RT, these areas need to receive the lowest possible dose while still maintaining adequate tumor coverage and sparing organs at risk. This can be achieved with techniques such as volumetric modulated arc therapy or proton therapy. The balance between treating the cancer and reducing the long-term effects of said treatment is something that needs to be explored in the future.

Limitations of this study include the limited number of scans suitable for analysis. As discussed in the results, only 28 out of a total of 206 glioma patients had scans of sufficient quality to perform proper and reliable cortical thickness measurements. While this rigorous selection ensures the best possible results, it does limit the power required to find small differences between the pre-RT and post-RT cortex.

Second, the administration of chemotherapy to the majority of patients could have an effect on the cortical thickness. While the agents administered in this study have not been linked to changes in brain morphology, studies in breast cancer and childhood leukemia show that certain chemotherapeutic agents have an effect on both white and gray matter dimentions.^46–49^ However, our study examined the relation between dose and thickness, and any effects of chemotherapy on the cortex are expected to be independent of radiation dose, as well as region independent. However, we cannot exclude the possibility that the observed differences are partly related to the administered chemotherapeutic agents. In a sensitivity analysis in which we corrected for chemotherapy, we found no major changes to the main results, suggesting that the role of chemotherapy to the observed effects is limited.

Also, in fitting a linear model to the dose–thickness relation, we may have overlooked any nonlinear relation between the two factors. It is important to note, however, that the exact relation between dose and cortical thinning is still not well understood. Therefore, unsupported, advanced models may result in spurious overfitting. With a robust and automated image processing pipeline, as well as statistical inference, we ensured to exclude false positive results.

Another possibility in this study is the introduced selection bias as we have selected patients with long enough survival, which may influence the results. However, we are interested in long-term effects of RT, which are of course only relevant to patients with long survival times. Another possible source of selection bias is the fact that we excluded patients with large cerebral damage beyond the PTV that could influence the automatic cortical thickness estimation. However, this occurred in only 3 patients, so we do not expect a big influence of this fact on the found results. This study also only examines glioma patients, which might mean that our results are not applicable to other primary and metastatic brain tumors.

Furthermore, the proximity of areas receiving higher dose to the tumor means that regional effects of the tumor microenvironment could have influenced the results. We, therefore, cannot rule out a tumor effect independent from the effect of radiation. However, as the area receiving dose is larger than the area of the tumor and as we have observed cortical thinning even at low doses, we expect this effect to not greatly influence our results.

Finally, the censoring pattern shown in Figure 4 could lead to overrepresentation of certain cortical regions in our data set. As the doses around the censored area and, thus, around the PTV, are highest, a dose–thickness relation is likely to be found in these regions. The censored areas are concentrated toward the frontal cortex, and two of our three identified regions are within areas of increased censoring.

To conclude, we have identified three areas susceptible to dose-dependent cortical thinning after radiation therapy. Should future studies conclude that irradiation of these areas lead to cognitive decline, they need to be spared in order to prevent this debilitating consequence of treatment.

## Funding

None

## Supplementary Material

vdaa060_suppl_Supplementary_Appendix

vdaa060_suppl_Supplementary_Figure_1

vdaa060_suppl_Supplementary_Figure_2

vdaa060_suppl_Supplementary_Figure_3

vdaa060_suppl_Supplementary_Figure_4

vdaa060_suppl_Supplementary_Table_1
