# Supplementary material for: Effect of radiation therapy on cerebral cortical thickness in glioma patients: Treatment-induced thinning of the healthy cortex
Source: Neurooncol Adv. 2020 May 21;2(1):vdaa060. doi: 10.1093/noajnl/vdaa060 (PMC7284116; doi:10.1093/noajnl/vdaa060)
Supplement: vdaa060_suppl_Supplementary_Appendix [file vdaa060_suppl_supplementary_appendix.docx]

**Appendix 1**

**Detailed methods**

Patient selection and data collection

We identified 28 patients (median age: 51, range: 19-71, 11 females) who were treated with RT for newly discovered grade II-IV glioma at the department of Radiation Oncology of our institution after January 2016. Patients were eligible for inclusion when the following criteria were met: treatment planning CT and MRI present, and of sufficient quality; survival < 270 days after RT; at least 1 follow-up MRI 270 days after RT present, and of sufficient quality. Clinical MRI and CT scans made for RT treatment planning were extracted from patient records, along with all follow-up MRIs, and clinical and demographic characteristics.

Image acquisition

For every patient the pre-RT CT and MRI were collected, as well as all available follow-up MRIs. All MR images were acquired on a 3T Philips Ingenia scanner (Philips Medical Systems, Best, The Netherlands) as part of routine clinical care. The imaging protocol is part of the standard brain MRI protocol of the department. T1-weighted MR images were acquired with a 3D turbo-spin echo (TSE) sequence without gadolinium enhancement with the following parameters: TR = 8.1 ms, TE = 3.7 ms, flip angle = 8°, 213 continuous axial slices without gap, matrix: 207 x 289, voxel resolution 1 ×0.96×0.96mm^3^.

The planning CT scans were acquired on a Brilliance Big bore scanner (Philips Medical Systems, Best, The Netherlands), with a tube potential of 120 kVp, using a matrix size of 512 × 512 and 0.65 × 0.65 × 3.0 mm voxel size.

Image processing

All data was processed with Statistical Parametric Mapping (SPM),^1^ Computational Anatomy Toolbox (CAT12)^2^ and in-house algorithms developed in MATLAB (Mathworks, Natick, Massachusetts).

First, the field-of-view (FOV) of the CT images were cropped to decrease computational load and to increase registration performance in later stages. As a result, the neck and the shoulders of the patients were excluded. The same cropping settings were applied for the dose and planning-clinical-gross target volume (TV) maps per patient. The PTV mask was slightly dilated with 3mm radius sphere with FSL^3^ utility tool *fslmaths*.

The T1 images were first rigidly co-registered to the mean image by an inverse-consistent realignment via SPM’s longitudinal rigid registration module. This is the only image processing step, when smoothing was introduced to the T1 images via resampling. The cropped CT image, as well as the associated dose and TV maps, was then registered to the mean T1 via SPM with an affine transformation using normalized mutual information as cost function. We used the mean T1 rather than the baseline, because the former had higher SNR, which lead to a better registration result overall. This step resulted that the RT images and the MRIs are in the same space. Next, the rigidly coregistered T1s were processed with CAT12’s segmentation pipeline using the full affine preprocessing package with the default settings, otherwise mentioned later. Briefly, the package includes bias-field inhomogeneity correction, spatial-adaptive non local means (SANLM) denoising^4^, tissue segmentation to grey matter (GM), white matter (WM) and cerebrospinal fluid (CSF)^5^ and spatially normalization using the DARTEL algorithm^6^. The segmentations were was further fine-tuned by accounting for partial volume effects^7^ by using a hidden Markov Random Field (MRF) model.^8^ The regions covered by the PTV were ignored from tissue classification. If the cerebral damage, e.g.: edema, extended beyond the PTV in either the baseline or the follow-up images than the affected subject was removed from the analysis.

Cortical thickness and central surfaces were estimated with CAT12 using the fully automated projection-based thickness (PBT) estimation method, no manual correction was performed. PBT includes correction for sulcal asymmetries and blurring. Topological correction is performed through an approach based on spherical harmonics.^9^ For inter-subject comparisons, cortical thickness maps were resampled^10^ to the Freesurfer^11^ surface template, which contains 164k mesh per hemisphere and were areal smoothed with a 15mm kernel size. Dose maps and dilated TV maps were mapped to the template surface too with an areal smoothing of 5mm kernel size. The spherical remapping resulted in that all individual features (cortical thickness, dose and PTV mask) were mapped to the same template surface, which allows vertexwise statistical comparison. The within-subject cortical thickness difference was calculated by subtracting the baseline and the follow-up cortical thickness surfaces in every vertex. In every subject the cortical thickness difference and the dose maps were censored with the surface resampled PTV maps to avoid spurious thickness-dose relations, which may originate from false thickness estimation approximating to the tumor. The censored vertexwise values were imputed with the local, cohort-mean of the cortical thickness and dose values. Previously, PBT was rigorously validated with spherical and brain phantoms representing wide range of settings (e.g.: noise, artifacts, etc.) and thickness. Furthermore, the CAT12 toolbox shows excellent test-retest reliability (R^2^ = 0.986) and PBT was tested against different cortical surface reconstruction methods resulted in fewer errors than comparable techniques.^12,13^

Statistical analysis

Vertexwise statistical comparison of cortical thickness change and dose correlation was carried out with a permutation test with 10000 iterations performed with the permutation analysis of linear models (PALM)^14–16^ toolbox version alpha104, a Matlab based open-source software package. We used nonparametric permutations as they proved efficient in diminishing false positive results when compared with parametric methods.^17^ Significance of a correlation was determined at p_corr_ < 0.05 using family-wise error rate (FWER) adjustment to correct for multiple comparisons and 2 dimensional Threshold-Free Cluster Enhancement (TFCE)^18^ was used to boost the statistical power. Tail approximation was used for faster calculations.^19^ Age at the time of the diagnosis and sex of the patients were included as nuisance regressors. The yearly rate of change per dose [in %/Gy/year] is reported to grade the practical significance of the results.^20^ A graphical overview of the image processing pipeline is shown in Fig. 2. The described methods completely fulfill the requirements of the most recent guideline in analyzing surface features of RT studies.^21^

**References**

1. Penny W, Friston K, Ashburner J, Kiebel S, Nichols T. *Statistical Parametric Mapping: The Analysis of Functional Brain Images*.; 2007. doi:10.1016/B978-0-12-372560-8.X5000-1

2. Gaser C, Dahnke R. CAT-a computational anatomy toolbox for the analysis of structural MRI data. *Hbm*. 2016;2016(7):336-348.

3. Jenkinson M, Beckmann CF, Behrens TEJ, Woolrich MW, Smith SM. FSL. *Neuroimage*. 2012;62(2):782-790. doi:10.1016/j.neuroimage.2011.09.015

4. Manjón J V., Coupé P, Martí-Bonmatí L, Collins DL, Robles M. Adaptive non-local means denoising of MR images with spatially varying noise levels. *J Magn Reson Imaging*. 2010;31(1):192-203. doi:10.1002/jmri.22003

5. Ashburner J, Friston KJ. Unified segmentation. *Neuroimage*. 2005;26(3):839-851. doi:10.1016/j.neuroimage.2005.02.018

6. Ashburner J. A fast diffeomorphic image registration algorithm. *Neuroimage*. 2007;38(1):95-113. doi:10.1016/j.neuroimage.2007.07.007

7. Tohka J, Zijdenbos A, Evans A. Fast and robust parameter estimation for statistical partial volume models in brain MRI. *Neuroimage*. 2004;23(1):84-97. doi:10.1016/j.neuroimage.2004.05.007

8. Cuadra MB, Cammoun L, Butz T, Cuisenaire O, Thiran J-P. Comparison and validation of tissue modelization and statistical classification methods in T1-weighted MR brain images. *IEEE Trans Med Imaging*. 2005;24(12):1548-1565. doi:10.1109/TMI.2005.857652

9. Yotter RA, Dahnke R, Thompson PM, Gaser C. Topological correction of brain surface meshes using spherical harmonics. *Hum Brain Mapp*. 2011;32(7):1109-1124. doi:10.1002/hbm.21095

10. Yotter RA, Thompson PM, Gaser C. Algorithms to Improve the Reparameterization of Spherical Mappings of Brain Surface Meshes. *J Neuroimaging*. 2011;21(2):e134-e147. doi:10.1111/j.1552-6569.2010.00484.x

11. Fischl B. FreeSurfer. *Neuroimage*. 2012;62(2):774-781. doi:10.1016/j.neuroimage.2012.01.021

12. Righart R, Schmidt P, Dahnke R, et al. Volume versus surface-based cortical thickness measurements: A comparative study with healthy controls and multiple sclerosis patients. Paul F, ed. *PLoS One*. 2017;12(7):e0179590. doi:10.1371/journal.pone.0179590

13. Seiger R, Ganger S, Kranz GS, Hahn A, Lanzenberger R. Cortical Thickness Estimations of FreeSurfer and the CAT12 Toolbox in Patients with Alzheimer’s Disease and Healthy Controls. *J Neuroimaging*. 2018;28(5):515-523. doi:10.1111/jon.12521

14. Winkler AM, Ridgway GR, Webster MA, Smith SM, Nichols TE. Permutation inference for the general linear model. *Neuroimage*. 2014;92:381-397. doi:10.1016/j.neuroimage.2014.01.060

15. Nichols T, Holmes A. Nonparametric Permutation Tests for Functional Neuroimaging. *Hum Brain Funct Second Ed*. 2003;25(August 1999):887-910. doi:10.1016/B978-012264841-0/50048-2

16. Holmes AP, Blair RC, Watson JD, Ford I. Nonparametric analysis of statistic images from functional mapping experiments. *J Cereb Blood Flow Metab*. 1996;16(1):7-22. doi:10.1097/00004647-199601000-00002

17. Eklund A, Nichols TE, Knutsson H. Cluster failure: Why fMRI inferences for spatial extent have inflated false-positive rates. *Proc Natl Acad Sci*. 2016;113(28):7900-7905. doi:10.1073/pnas.1602413113

18. Smith SM, Nichols TE. Threshold-free cluster enhancement: Addressing problems of smoothing, threshold dependence and localisation in cluster inference. *Neuroimage*. 2009;44(1):83-98. doi:10.1016/j.neuroimage.2008.03.061

19. Winkler AM, Ridgway GR, Douaud G, Nichols TE, Smith SM. Faster permutation inference in brain imaging. *Neuroimage*. 2016;141:502-516. doi:10.1016/j.neuroimage.2016.05.068

20. Wasserstein RL, Lazar NA. The ASA Statement on p -Values: Context, Process, and Purpose. *Am Stat*. 2016;70(2):129-133. doi:10.1080/00031305.2016.1154108

21. Nagtegaal SHJ, David S, van der Boog ATJ, Leemans A, Verhoeff JJC. Changes in cortical thickness and volume after cranial radiation treatment: A systematic review. *Radiother Oncol*. 2019;135:33-42. doi:10.1016/j.radonc.2019.02.013
