# Supplementary material for: Effect of radiation therapy on cerebral cortical thickness in glioma patients: Treatment-induced thinning of the healthy cortex
Source: Neurooncol Adv. 2020 May 21;2(1):vdaa060. doi: 10.1093/noajnl/vdaa060 (PMC7284116; doi:10.1093/noajnl/vdaa060)
Supplement: vdaa060_suppl_Supplementary_Figure_1 [file vdaa060_suppl_supplementary_figure_1.pptx]

## Slide 1
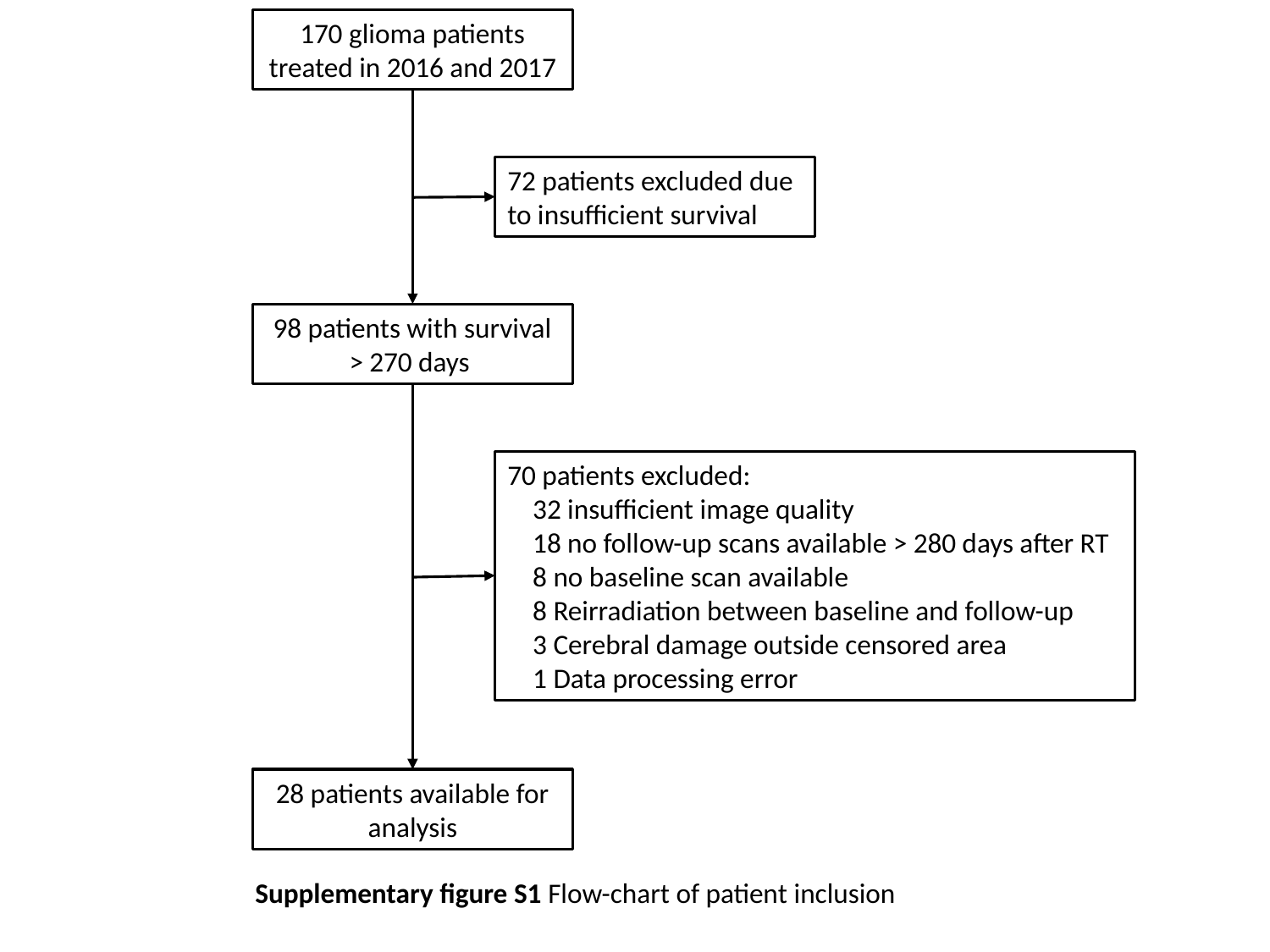

170 glioma patients treated in 2016 and 2017
72 patients excluded due to insufficient survival
98 patients with survival > 270 days
70 patients excluded:
32 insufficient image quality
18 no follow-up scans available > 280 days after RT
8 no baseline scan available
8 Reirradiation between baseline and follow-up
3 Cerebral damage outside censored area
1 Data processing error
28 patients available for analysis
Supplementary figure S1 Flow-chart of patient inclusion
