# Supplementary material for: Effect of radiation therapy on cerebral cortical thickness in glioma patients: Treatment-induced thinning of the healthy cortex
Source: Neurooncol Adv. 2020 May 21;2(1):vdaa060. doi: 10.1093/noajnl/vdaa060 (PMC7284116; doi:10.1093/noajnl/vdaa060)
Supplement: vdaa060_suppl_Supplementary_Figure_2 [file vdaa060_suppl_supplementary_figure_2.pdf]

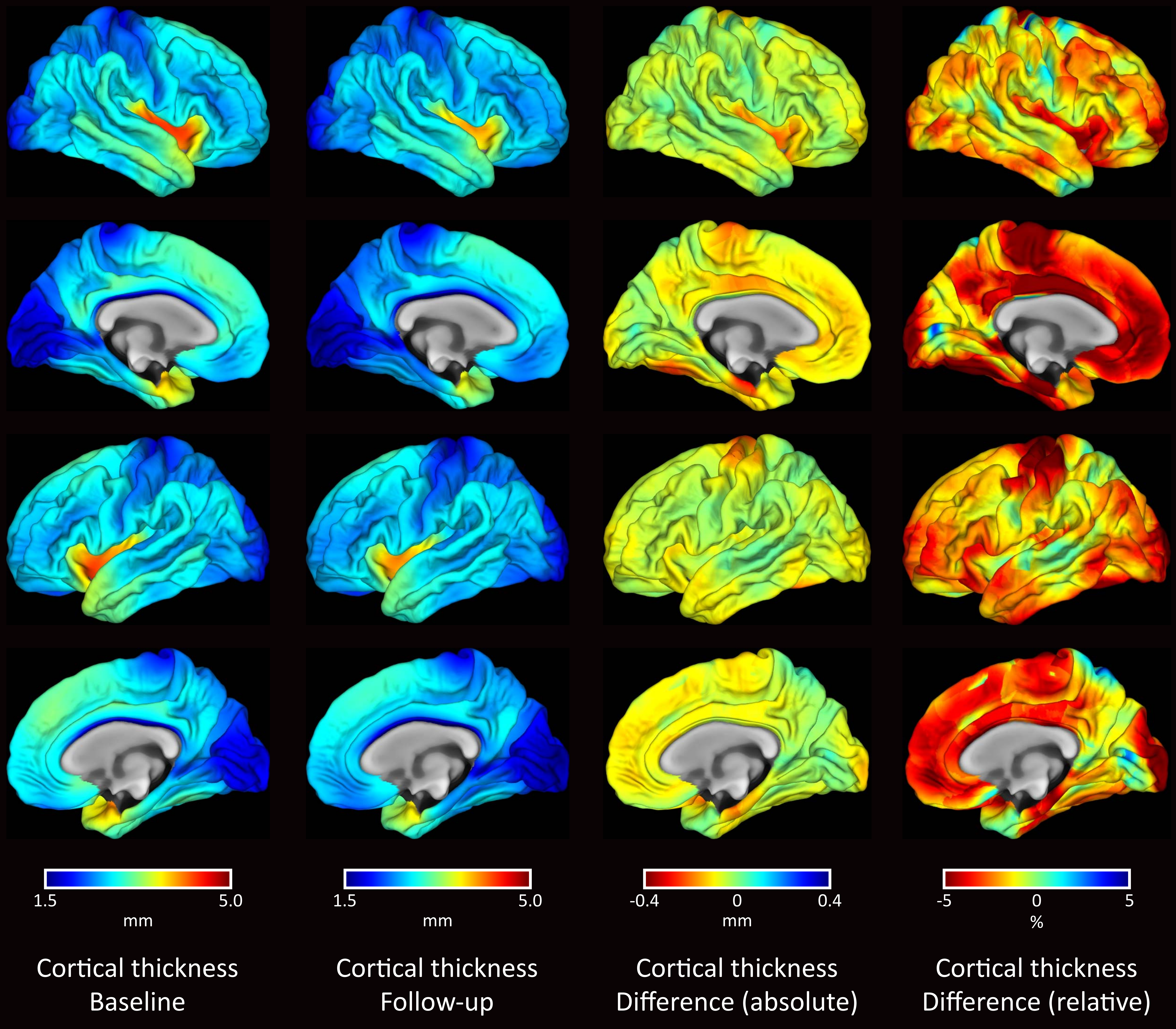

Supplementary figure 2 Mean local cortical thickness at baseline and follow-up, and mean absolute and relative differences in cortical thickness
