# Supplementary figures and images for: Effect of radiation therapy on cerebral cortical thickness in glioma patients: Treatment-induced thinning of the healthy cortex
Source: Neurooncol Adv. 2020 May 21;2(1):vdaa060. doi: 10.1093/noajnl/vdaa060 (PMC7284116; doi:10.1093/noajnl/vdaa060)

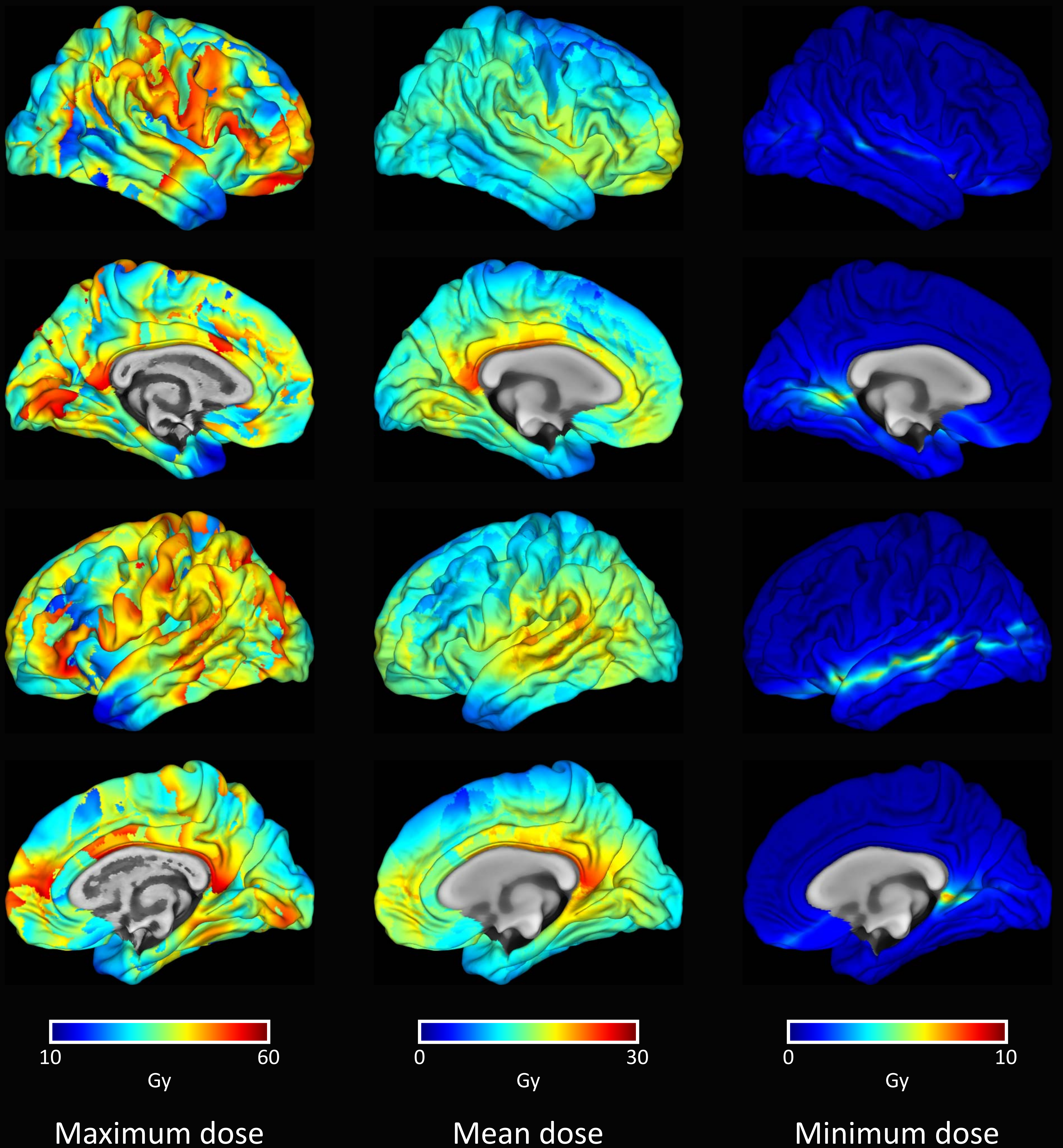

**Supplementary figure 3** Local maximum, mean and minimum dose, after PTV censoring

Supplement: vdaa060_suppl_Supplementary_Figure_3 [file vdaa060_suppl_supplementary_figure_3.pdf]

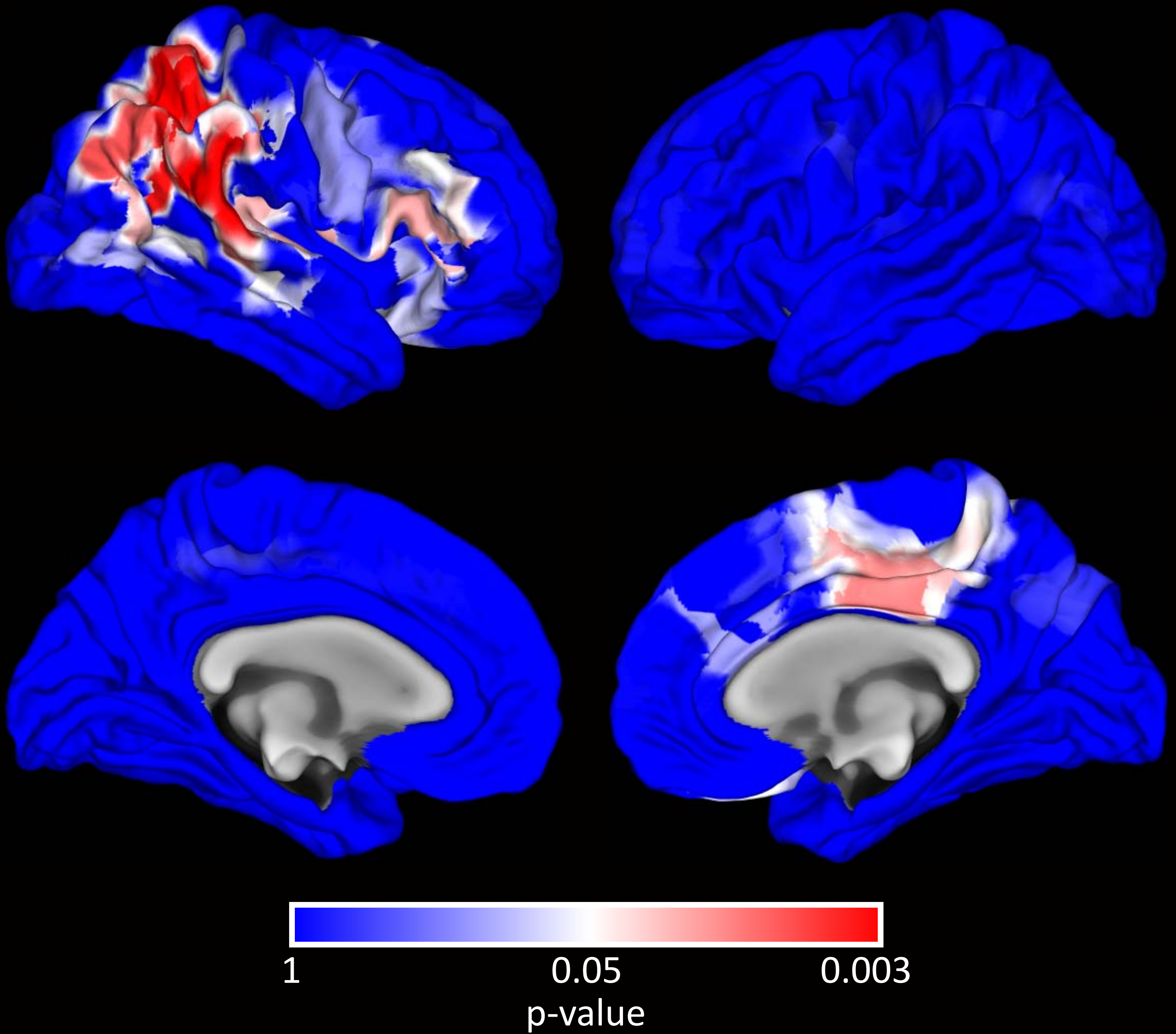

**Supplementary figure 4** Local p-value of relation between dose and cortical thinning

Supplement: vdaa060_suppl_Supplementary_Figure_4 [file vdaa060_suppl_supplementary_figure_4.pdf]
