# Supplementary material for: Effect of radiation therapy on cerebral cortical thickness in glioma patients: Treatment-induced thinning of the healthy cortex
Source: Neurooncol Adv. 2020 May 21;2(1):vdaa060. doi: 10.1093/noajnl/vdaa060 (PMC7284116; doi:10.1093/noajnl/vdaa060)
Supplement: vdaa060_suppl_Supplementary_Table_1 [file vdaa060_suppl_supplementary_table_1.docx]

| **Supplementary Table 1** Areas of significant dose-dependent cortical thinning in the right hemisphere, correcting for chemotherapy administration. | | | | | |
| --- | --- | --- | --- | --- | --- |
| **Cluster** | **Cluster size (no of vertices)** | **Mean cortical thickness change (µm/Gy/year)** | **Mean cortical thickness change (%/Gy/year)** | **p-value*** | **Corresponding atlas regions**** |
| 1 | 22312 | -5 | -0.19 | 0.017 | Inferior parietal (24.9%) |
|  |  |  |  |  | Supramarginal (20.7%) |
|  |  |  |  |  | Superior parietal (19.9%) |
|  |  |  |  |  | Superior temporal (10.9%) |
|  |  |  |  |  | Pars triangularis (5.5%) |
|  |  |  |  |  | Pars opercularis (4.8%) |
|  |  |  |  |  | Postcentral (3.4%) |
|  |  |  |  |  | Transverse temporal (3.3%) |
|  |  |  |  |  | Insula (2.5%) |
|  |  |  |  |  | Precentral (2.2%) |
|  |  |  |  |  | Bank of STS (2.0%) |
|  | | | | | |
| 2 | 3376 | -6 | -0.23 | 0.029 | Posterior cingulate (56.7%) |
|  |  |  |  |  | Paracentral (28.8%) |
|  |  |  |  |  | Superior frontal (14.6%) |
|  | | | | | |
| 3 | 612 | -6 | -0.23 | 0.044 | Paracentral (55.7%) |
|  |  |  |  |  | Precuneus (44.3%) |
|  | | | | | |
| 4 | 250 | -26 | -0.76 | 0.046 | Lateral orbital frontal (96.8%) |
|  |  |  |  |  | Medial orbital frontal (3.2%) |
| * Corrected for multiple testing with family-wise error rate adjustment  ** According to Desikan-Killiany brain atlas^29^ | | | | | |
